# Nonunion Fractures: Trends in Epidemiology and Treatment of Femur Fractures, 2017-2022

**DOI:** 10.7759/cureus.70566

**Published:** 2024-09-30

**Authors:** Kassem Ghayyad, Pascal Escobar, Tyler F Beaudoin, Luke Wandersleben, Michael Hawks, Atif Ahmed, Amir R Kachooei

**Affiliations:** 1 Orthopaedic Surgery, Rothman Orthopaedics Florida at AdventHealth, Orlando, USA; 2 Orthopaedic Surgery, University of Maryland School of Medicine, Baltimore, USA; 3 Orthopaedic Surgery, Loma Linda University School of Medicine, Orlando, USA; 4 Orthopaedics, University of Central Florida, Orlando, USA; 5 Orthopedics, Orthopedic Research Center, Mashhad University of Medical Sciences, Mashhad, IRN

**Keywords:** intramedullary nailing (imn), non-union shaft femur, open reduction and internal fixation (orif), socio-demographic factors, trauma epidemiology

## Abstract

Background: The incidence of femur fractures has increased in recent years, along with the rate of surgery and subsequent nonunion following these fractures. Nonunion is a significant concern due to the burden it places on both patients and the healthcare system. This study aims to investigate the demographic factors associated with femoral shaft fracture nonunion by comparing two surgical management approaches: open reduction internal fixation (ORIF) with plating versus closed reduction and fixation using intramedullary nailing (IMN).

Methods: The TriNetX database was queried using current procedural terminology (CPT) codes for surgical repair of femoral shaft fractures (FSFs), including IMN surgery (CPT: 27506) and femur ORIF (CPT: 27507). The subsequent chronological nonunion cases were identified using the 10th revision of the International Classification of Diseases (ICD-10) codes for femoral nonunion (S72.301K, S72.302K). Results were analyzed both descriptively and comparatively to assess differences among patients. Factors considered included age, sex, ethnicity, race, smoking status, and the type of surgical management (ORIF versus nailing) across the six-year study period.

Results: From 2017 to 2022, the prevalence of femoral shaft fracture surgeries increased for both IMN and ORIF. The rate of nonunion was 2.1% following IMN and 1.7% following ORIF. The mean age for nonunion was 54 years (SD: 20) following IMN and 63 years (SD: 18) following ORIF. Patients with a positive smoking history had higher rates of nonunion compared to those without.

Conclusion: Our study revealed a relatively consistent rate of FSFs and nonunion over the six-year period. However, with a growing population, the absolute number of cases is steadily increasing, underscoring the burden on the healthcare system. This study contributes to the growing body of literature focused on improving patient outcomes and promoting health equity in fracture management practices.

## Introduction

As the population of the United States continues to grow and age, the risk of fractures increasingly becomes a significant health concern [1]. In the United States, femoral shaft fractures (FSFs) occur at a rate of 9.5 to 19 per 100,000 individuals annually [2]. Among the elderly, the incidence of FSFs rises to about 19.9 per 100,000 [3]. One of the most concerning complications following an FSF is femur fracture (FF) nonunion (FFN). A fracture is typically classified as a nonunion if the fractured bone has not united nine months after the initial injury [4]. Current estimates suggest that FFN occurs in 1.9-5% of FSFs [5]. Patients with nonunion often experience significant burdens, including pain, loss of function, and psychological distress [6]. Understanding the association between various risk factors for fractures, nonunion, and the need for nonunion surgery is critical for improving patient outcomes and reducing the burden on those affected by these conditions.

FSFs display different distributions based on age and gender. High-energy trauma is a more common cause of FSF in men and younger patients, while women and older individuals are more likely to experience FSFs from ground-level falls [7]. For many populations, particularly the elderly, FFs can mark a significant change in daily life, often preventing patients from continuing their activities of daily living independently. This is especially concerning because falls, which often result in FFs, are the leading cause of morbidity and disability among the elderly [8]. However, discrepancies in outcomes following FFs are not limited to age and gender. Several studies have shown that demographic factors such as ethnicity, socioeconomic status, and underlying health conditions also affect the prognosis for fracture healing [9-11].

As with all fractures, nonunion is a concerning complication of FSFs. This study aims to leverage the TriNetX database, an extensive global network, to enhance our understanding of the frequency and demographic variables associated with FF surgeries, including intramedullary nailing (IMN) and open reduction internal fixation (ORIF) with plating, and the subsequent occurrence of nonunion.

## Materials and methods

A retrospective study using the TriNetX Global Collaborative Network was conducted and queried on December 1, 2023. The data collection methodology does not include patient-identifiable information, making it exempt from Institutional Review Board (IRB) approval.

Patient cohorts were defined by the type of surgery using the current procedural terminology (CPT) codes for femur IMN surgery (CPT: 27506) and femur ORIF with plating (CPT: 27507). The subsequent occurrence of nonunion was identified using the 10th revision of the International Classification of Diseases (ICD-10) codes for femur nonunion (S72.301K, S72.302K). All patients who underwent FF fixation between January 1, 2017, and December 31, 2022, were included. Smoking history (Z87.891) was also explored.

Demographic features

Patient information was extracted, including age, sex, ethnicity, race, and smoking status. Age was categorized into four groups: 0-17, 18-39, 40-64, and 65-90. Ethnicity was divided into Hispanic or Latino, Not Hispanic or Latino, and Unknown. Race was categorized as White, Black, Asian, Native American or Pacific Islander, American Indian or Alaska Native, and Unknown.

Statistical analysis

Since sample sizes below 10 are not retrievable with the query, we excluded the Native Hawaiian/Pacific Islander and American Indian/Alaska Native racial groups from the incidence rate calculations due to low sample sizes. Patients of unknown sex, ethnicity, and race were similarly excluded. Low sample sizes also led to the exclusion of Asian individuals and individuals aged 0-17 years from surgery calculations. A chi-squared test was performed to assess significant differences between the groups.

Global collaborative network

The TriNetX Global Collaborative Network is a Web-based database that facilitates research on population cohorts, feasibility queries, and collaboration with medical researchers worldwide. The database contains over 400 million de-identified patient records that can be accessed on demand without prior IRB approval. It provides access to patient demographics, diagnoses, procedures, labs, and medications. Data are sourced from collaborations between over 200 community and academic-based healthcare organizations and industry partners worldwide.

## Results

Femoral IMN fixation 

From 2017 to 2022, the rate of femoral IMN fixation showed an upward trend. The mean age of patients was 48 years (SD: 25) and remained consistent throughout the study period, with only slight variations (Table 1).

**Table 1:** Demographic characteristics of the patients underwent femur shaft IMN (CPT: 27506) from 2017 to 2022 IMN: intramedullary nail, CPT: current procedural terminology, SD: standard deviation, Hx: history.

Category	2017 (N = 2114)	2018 (N = 2386)	2019 (N = 2421)	2020 (N = 2580)	2021 (N = 3143)	2022 (N = 3294)	2017-2022 (N = 15,938)
Age							
Mean age ± SD	48.5 ± 24.8	47.7 ± 24.6	48.1 ± 34.7	46.7 ± 24.4	46 ± 25.2	47.9 ± 25.4	47.5 ± 25
0-17	199 (9.4%)	230 (9.6%)	214 (8.8%)	201 (7.8%)	344 (10.9%)	326 (9.9%)	1514 (9.5%)
18-39	787 (37.2%)	890 (37.3%)	907 (37.5%)	1040 (40.3%)	1174 (37.4%)	1172 (35.6%)	5970 (37.5%)
40-64	464 (22.0%)	532 (22.3%)	529 (21.9%)	581 (22.5%)	712 (22.7%)	715 (21.7%)	3533 (22.2%)
65-90	664 (31.4%)	734 (30.8%)	771 (31.9%)	758 (29.4%)	913 (29.1%)	1081 (32.8%)	4921 (30.9%)
Sex							
Female	783 (37.0%)	947 (39.7%)	991 (40.9%)	997 (38.6%)	1230 (39.1%)	1366 (41.5%)	6314 (39.6%)
Male	1185 (56.1%)	1308 (54.8%)	1284 (53.0%)	1467 (56.9%)	1697 (54.0%)	1719 (52.2%)	8660 (54.3%)
Unknown	130 (6.2%)	123 (5.2%)	139 (5.7%)	126 (4.9%)	144 (4.6%)	168 (5.1%)	830 (5.2%)
Ethnicity							
Not Hispanic or Latino	1584 (74.9%)	1795 (75.2%)	1783 (73.7%)	1993 (77.3%)	1383 (44.0%)	2524 (76.6%)	11062 (69.4%)
Unknown	263 (12.4%)	279 (11.7%)	353 (14.6%)	337 (13.1%)	395 (12.6%)	441 (13.4%)	2068 (13.0%)
Hispanic or Latino	251 (11.9%)	304 (12.7%)	278 (11.5%)	260 (10.1%)	293 (9.3%)	288 (8.7%)	1674 (10.5%)
Race							
White	1317 (62.3%)	1524 (63.9%)	1538 (63.5%)	1571 (60.9%)	1786 (56.8%)	1963 (59.6%)	9699 (60.9%)
Unknown	253 (12.0%)	274 (11.5%)	292 (12.1%)	304 (11.8%)	359 (11.4%)	381 (11.6%)	1863 (11.7%)
Black	424 (20.1%)	428 (17.9%)	461 (19.0%)	578 (22.4%)	725 (23.1%)	683 (20.7%)	3299 (20.7%)
Asian	38 (1.80%)	54 (2.3%)	48 (2.0%)	44 (1.7%)	59 (1.9%)	73 (2.2%)	316 (2.0%)
Smoking Hx (z87.891)							
Yes	328 (15.5%)	362 (15.2%)	401 (16.6%)	411 (15.9%)	438 (13.9%)	490 (14.9%)	2430 (15.3%)
No	1786 (84.5%)	2024 (84.8%)	2020 (83.4%)	2169 (84.1%)	2705 (86.1%)	2804 (85.1%)	13508 (84.8%)

Nonunion following femoral IMN fixation 

The overall rate of nonunion following IMN for FSFs was 2.1%, remaining relatively constant over the study period. The rate was highest in 2019 at 2.7% and lowest in 2021 at 1.7%. The mean age of patients with nonunion following femur IMN was 54 ± 20 years. The male-to-female ratio was approximately 1.45:1 (Table 2).

**Table 2:** Demographic characteristics of the patients with nonunion following femur shaft IMN (S72.301K, S72.302K) IMN: intramedullary nail; N: Number of patients.

Category	2017 (N = 45)	2018 (N = 48)	2019 (N = 65)	2020 (N = 61)	2021 (N = 54)	2022 (N = 60)	2017-2022 (N = 333)
IMN nonunion percentage (%)	2.10%	2.00%	2.70%	2.40%	1.70%	1.80%	2.10%
Mean age (SD)	57 (19)	55 (19)	55 (20)	52 (20)	53 (20)	50 (19)	54 (20)
Sex							
Female	24 (53.3%)	17 (35.4%)	26 (40.0%)	23 (37.7%)	17 (31.5%)	25 (41.7%)	132 (39.6%)
Male	21 (46.7%)	31 (64.6%)	29 (44.6%)	38 (62.3%)	37 (68.5%)	35 (58.3%)	191 (57.4%)

FSF ORIF 

The overall rate of ORIF following an FSF was 7.8%. The percentage of patients undergoing femur ORIF remained stable at around 8% throughout the study period, with a slight drop to 7% in 2020. The mean age of patients remained consistent, ranging from 63 to 65 years, with an overall average of 64 ± 28 years (Table 3).

**Table 3:** Demographic characteristics of the patients underwent femur shaft ORIF (CPT: 27507) between 2017 to 2022 SD: standard deviation, Hx: history, ORIF: open reduction and internal fixation, CPT: current procedural terminology.

Category	2017 (N = 547)	2018 (N = 608)	2019 (N = 633)	2020 (N = 630)	2021 (N = 775)	2022 (N = 790)	2017-2022 (N = 3983)
Plate percentage (%)	8%	8%	8%	7%	8%	8%	8%
Age							
Mean age ± SD	64.5 ± 27.4	64.3 ± 28.1	65.4 ± 26.4	64.2 ± 27	62.5 ± 28.1	63.7 ± 27.6	64 ± 27.5
0-17	66 (12.1%)	81 (13.3%)	86 (13.6%)	88 (14.0%)	126 (16.3%)	119 (15.1%)	566 (14.2%)
18-39	59 (10.8%)	67 (11.0%)	37 (5.8%)	46 (7.3%)	55 (7.1%)	49 (6.2%)	313 (7.9%)
40-64	61 (11.2%)	58 (9.5%)	78 (12.3%)	73 (11.6%)	106 (13.7%)	101 (12.8%)	477 (12.0%)
65-90	361 (66.0%)	402 (66.1%)	432 (68.2%)	423 (67.1%)	488 (63.0%)	521 (65.9%)	2627 (66.0%)
Sex							
Female	296 (54.1%)	315 (51.81%)	337 (53.24%)	323 (51.27%)	423 (54.58%)	411 (52.03%)	2105 (52.85%)
Male	202 (36.9%)	242 (39.80%)	238 (37.60%)	250 (39.68%)	289 (37.29%)	314 (39.75%)	1535 (38.54%)
Unknown	49 (9.0%)	52 (8.6%)	58 (9.2%)	57 (9.0%)	63 (8.1%)	65 (8.2%)	344 (8.6%)
Ethnicity							
Not Hispanic or Latino	414 (75.7%)	450 (74.0%)	484 (76.5%)	484 (76.8%)	625 (80.6%)	625 (79.1%)	3082 (77.4%)
Hispanic or Latino	39 (7.1%)	45 (7.4%)	29 (4.6%)	29 (4.6%)	44 (5.7%)	35 (4.4%)	221 (5.5%)
Race							
White	425 (77.7%)	456 (75.0%)	479 (75.7%)	461 (73.2%)	574 (74.1%)	598 (75.7%)	2993 (75.1%)
Black	39 (7.1%)	48 (7.9%)	44 (7.0%)	51 (8.1%)	75 (9.7%)	65 (8.2%)	322 (8.1%)
Asian	10 (1.8%)	10 (1.6%)	10 (1.6%)	14 (2.2%)	10 (1.3%)	10 (1.3%)	64 (1.6%)
Smoking Hx (z87.891)							
Yes	104 (19.0%)	129 (21.2%)	162 (25.6%)	141 (22.4%)	177 (22.8%)	185 (23.4%)	898 (22.5%)
No	443 (81.0%)	479 (78.8%)	471 (74.4%)	489 (77.6%)	598 (77.2%)	605 (76.6%)	3085 (77.4%)

FSF ORIF nonunion 

The overall rate of nonunion following ORIF for FSFs was 1.7%. The mean age of patients with nonunion after femur ORIF was 63 ± 18 years. Of the 67 patients, 41 (61%) were female and 26 (39%) were male, with an approximate female-to-male ratio of 1.58:1 (Table 4).

**Table 4:** Demographic characteristics of the patients with nonunion following femur shaft ORIF (S72.301K, S72.302K) SD: standard deviation, ORIF: open reduction and internal fixation.

Category	2017-2022 (N = 67)
Plate nonunion percentage	1.7%
Mean age ± SD	63 ± 18
Sex	
Female	41 (61.2%)
Male	26 (38.8%)

## Discussion

Recognizing demographic factors that influence health outcomes is vital for improving patient care and targeting interventions to enhance health equity for at-risk groups. This study provides valuable insights into the incidence of FSFs, the demographic factors associated with fracture nonunion, and the surgical management of nonunion.

FSFs are primarily managed through surgical intervention, with antegrade IMN being the gold standard treatment due to its high union rate, low complication rate, and increased fracture stability [7,12]. Previous studies have shown that only a minority of FSFs are treated conservatively, with such management often being temporary or dictated by the patient’s age and stability [12,13]. Although older patients tend to have a higher incidence of nonoperative management, less than 4% of patients over the age of 60 receive conservative treatment [14]. The increased prevalence of surgical management can be attributed to advancements in surgical techniques, implants, and a growing emphasis on early mobilization and rehabilitation [15].

The trend toward increased surgical fixation offers several advantages, including faster recovery, reduced risk of complications, improved functional outcomes, and lower mortality [7]. Nonunion rates are significantly lower in patients treated surgically compared to those treated conservatively. Studies on nonunion have found that the rate following IMN for FSF ranges from 1.1% to 14% [16]. While IMN treatment boasts a healing rate as high as 95% for FSF and plating treatment slightly lower at 91.6%, nonunion remains the most common complication for both surgical techniques [16,17]. Furthermore, nonunion rates are thought to be rising with the increased survival of patients with severe injuries [18]. Several factors can affect the healing process following FSF and its surgical management, and this study aimed to identify significant differences in patient outcomes based on age, sex, ethnicity, race, and smoking status.

In terms of patient age, the prevalence of FSFs was highest among the oldest age group, 65-90. Previous studies have shown that fractures in older patients are more likely to result in fatality [19,20]. As a result, FSFs in the elderly are less likely to be diagnosed as nonunion if mortality occurs before the fracture can be classified. Among elderly patients, the one-year mortality rate following FSFs is around 25%, compared to 10% in younger and middle-aged adults [14,21,22]. Although the elderly group (65-90) comprised the largest proportion (66%) of femoral plate operative management, this group ranked second to the 18-39 age group for IMN procedures. The differences in surgery incidence could be attributed to the increased surgical risks associated with older patients [9]. Despite a lower IMN surgical rate of 31% for the 65-90 age group, it was not significantly different from the 38% rate observed in the 18-39 age group.

Until 2019, the annual number of FSFs increased among all groups. However, between 2019 and 2020, the number of fractures did not change significantly, indicating a decrease in the rate of change seen until this point. Other studies also found a decrease in fractures associated with the initial COVID-19 lockdown in early 2020 [23-25]. One explanation for this decline could be reduced outdoor activities and fewer opportunities for accidents prone to causing fractures during the COVID-19 pandemic [26]. Despite the lower incidence of FSFs in 2019 and 2020, the incidence of nonunion and surgical management remained relatively stable for younger age groups (0-17 and 18-39). In contrast, older age groups (40-64 and 65-90) saw a decrease in nonunion and surgical management for FSFs. One potential explanation is that FFs are typically treated surgically. However, during the pandemic, the risk of COVID-19 infection in hospitals, reduced operating room availability, and the reluctance of elderly patients to enter high-risk healthcare settings likely reduced surgical management in this age group [27-30]. The increased mortality associated with FFs in older patients may have also contributed to the observed decrease in nonunion and surgical management between 2019 and 2020.

Previous findings indicate that the incidence of FFs increases with age, particularly in individuals over 80, most of whom are female [31]. Numerous studies show that as females age, their risk of bone fractures increases significantly, with higher rates of osteoporosis and fractures compared to men. Decreasing levels of estrogen associated with aging have been shown to reduce bone density in females, thus exacerbating the risk of fractures [32,33].

In this study, the prevalence of FSFs was consistently higher for females than for males. Even though fractures are more prevalent in females, the incidence of nonunion was significantly greater in males. However, the incidence of surgical management for nonunion was similar between the two groups. Among the current literature, it is speculated that male gender may increase nonunion risk, primarily due to gender-specific activity types and injury patterns [11]. This study compared female and male outcomes but did not subcategorize the genders into different age groups. Further research could compare gender outcomes by age, potentially using pre- and post-menopausal women as distinct groups for analysis.

With regard to race, FSFs were more prevalent among White patients compared to Black and Asian patients, with White individuals having approximately five times the prevalence of FSFs compared to Black patients. Several reports suggest that genetic factors may make White patients more susceptible to osteoporosis and lower bone mineral density (BMD), with some studies identifying genetic variants associated with European ancestry as predictive of fracture risk [34,35]. However, after adjusting for U.S. demographic distributions, this study found that FSF prevalence was slightly higher among Black patients.

Additionally, the incidence of nonunion was significantly higher among White patients compared to Black patients, while Asian patients had a significantly greater incidence of FSF nonunion than both Black and White patients. Similar studies have reported that Asian patients have a higher incidence of atypical femur fractures (AFFs) compared to White patients, with AFFs showing higher rates of nonunion and reoperations compared to FFs [10,36,37,38]. These findings align with previous reports indicating that Asian individuals experience the highest nonunion rates. Furthermore, this study found similar rates of operative management of FSFs across all racial groups considered. Overall, these results are surprising, considering that previous research has indicated that patients from minority groups tend to experience worse health outcomes [39,40].

Although the prevalence of FSFs was highest among non-Hispanic or Latino individuals in this study, Hispanic and Latino individuals had a significantly higher incidence of receiving surgical management. Since 2000, some studies have reported a decrease in the incidence of certain fractures among White patients, but not among Black, Asian American, and Hispanic populations [41,42]. Prompt surgical fixation of FSFs and other FFs is associated with better outcomes. However, Vazquez et al. found that non-White patients and those with lower socioeconomic status (SES) had worse outcomes following an FF [43]. In the United States, Black and Hispanic populations, who often have lower SES, experience worse outcomes following FFs due to a direct relationship between their SES and post-operative care, leading to higher rates of nonunion and further surgical management [44]. Considering these factors may help predict fracture, nonunion, and surgical management rates in minority groups.

Multiple studies have found that comorbidities such as cigarette smoking significantly slow the healing process of nonunion and extend the time to union [6,45,46]. Smoking is a known risk factor for both nonunion and premature death, which may underrepresent the cases of FSFs that would have developed into nonunion [47]. The association between smoking and fracture outcomes is particularly concerning, considering that about 12% of U.S. adults smoked cigarettes in 2021 [48].

Data from 59 healthcare organizations were queried using the TriNetX database, yielding a diverse patient population and reducing the likelihood of confounding factors. However, this study has several limitations, including the exclusion of certain racial groups due to small sample sizes and the lack of data on injury severity and fracture patterns. Additionally, the data were collected over only five years, limiting its applicability to long-term epidemiological trends. The TriNetX dataset, derived directly from electronic health record (EHR) systems of multiple healthcare organizations, may introduce variability due to differences among organizations and includes data only from select institutions, excluding patients with FSFs or FSF nonunion outside this network. As a result, the findings do not account for factors such as the mechanism of injury, underlying comorbidities, baseline functional status, or patient outcomes. Therefore, the results should not be used as a basis for therapy or treatment recommendations.

The findings of this study indicate that outcomes with conventional plates are comparable to those with IMN for the treatment of FSFs. Additionally, the results show that nonunion rates have marginally decreased, with IMN demonstrating an average nonunion rate of 2.1% and femur plate surgeries showing a rate of 1.7%. Appropriate surgical indications can reduce the risk of nonunion and its associated negative impacts on patients’ quality of life and healthcare systems. This study focused primarily on outcomes of FSFs but did not account for the extent or severity of the fractures. Future studies should aim to investigate potential risk factors for nonunion and surgical management of FSFs, particularly with respect to fracture type and severity. Additional factors, such as body mass index and injury severity, should also be further explored. Previous research has shown varying nonunion rates depending on the surgical technique used for fracture fixation. For instance, IMN has been associated with the highest union rates, especially when compared to screw plate fixation [7,21]. Additionally, nail exchange has been shown to be superior even in cases of nonunion [49]. Future analyses could compare the incidence of nonunion of the femoral shaft based on surgical techniques and demographic variables.

## Conclusions

This study observed consistent FSF and nonunion rates over six years, with nonunion rates of 1.7% for IMN and 2.1% for ORIF. However, due to the growing population in the United States, the absolute number of surgeries has steadily increased. Although nonunion rates for both ORIF and IMN were low, the rise in surgical management may influence these observed rates. Future research into nonunion risk factors and surgical management, particularly considering fracture type and severity, could help identify optimal treatment strategies to further reduce FSF nonunion rates.
